# Novel concept for neutron detection: proportional counter filled with ^10^B nanoparticle aerosol

**DOI:** 10.1038/srep41699

**Published:** 2017-02-09

**Authors:** F. D. Amaro, C. M. B. Monteiro, J. M. F. dos Santos, A. Antognini

**Affiliations:** 1 LIBPhys, Physics Department, University of Coimbra, P-3004-516 Coimbra, Portugal; 2 Institute for Particle Physics, ETH Zurich, 8093 Zurich, Switzerland; 3 Paul Scherrer Institute, 5232 Villigen-PSI, Switzerland

## Abstract

The high neutron detection efficiency, good gamma-ray discrimination and non-toxicity of ^3^He made of proportional counters filled with this gas the obvious choice for neutron detection, particularly in radiation portal monitors (RPM), used to control the illicit transport of nuclear material, of which neutron detectors are key components. ^3^He is very rare and during the last decade this gas has become increasingly difficult to acquire. With the exception of BF_3_, which is toxic, no other gas can be used for neutron detection in proportional counters. We present an alternative where the ^3^He atoms are replaced by nanoparticles made of another neutron sensitive material, ^10^B. The particles are dispersed in a gaseous volume, forming an aerosol with neutron sensitive properties. A proportional counter filled with such aerosol was exposed to a thermal neutron beam and the recorded response indicates that the neutrons have interacted with the particles in the aerosol. This original technique, which transforms a standard proportional gas mixture into a neutron sensitive aerosol, is a breakthrough in the field of radiation detection and has the potential to become an alternative to the use of ^3^He in proportional counters.

Proportional counters filled with ^3^He are the golden standard for thermal neutron detection. This gas has high neutron detection efficiency, good gamma-ray discrimination, is non-toxic and, until recently, was an accessible material^1^,^2^,^3^. These characteristics made of ^3^He proportional counters the obvious choice for neutron detection, particularly in radiation portal monitors (RPM), used to control the illicit transport of nuclear material, of which thermal neutron detectors are key components. ^3^He is, however, very rare and mostly a by-product of nuclear warhead production. With the large deployment of RPM in the last decade this gas has become increasingly difficult to acquire, with skyrocketing prices^1^,^2^. Besides domestic security, other applications where ^3^He is used, such as large area detectors in neutron research facilities, nuclear reactor control, cryogenic systems, well logging and medical imaging have also suffered from the reduced availability of this material^4^. With the exception of BF_3_, which is toxic and not suitable for applications outside the laboratory, no other gas exists that can be used for neutron detection in gaseous proportional counters.

Thermal neutron (n) detection in gaseous proportional counters is achieved via reactions of the type:


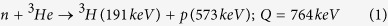



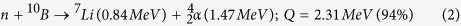


Due to momentum conservation, the products are emitted in opposite directions, each carrying a fraction of the total energy released (*Q*). The tagging of the thermal neutron is done by measuring the energy of the products released in the above reactions. These will travel in the proportional gas, ionizing its molecules and creating a track of ion-electron pairs, which number is proportional to the energy deposited in the gas. An electric field established inside the proportional counter multiplies the primary-electrons, being the resulting charge collected at an appropriate electrode. In ^3^He proportional counters another filling gas can be added to facilitate the collection of the reaction products energy: ^3^He acts as the stopping medium for the thermal neutrons via reaction (1) while the tritium and the proton are stopped by the filling gas, with higher stopping power than ^3^He. In large detectors both products of (1) fully deposit their energy in the proportional gas and the amplitude of the output pulses follows a Gaussian distribution with an average value proportional to *Q* (Fig. 1)^5^.

One of the alternatives to ^3^He is the Boron-lined proportional counter^6^,^7^,^8^ where the neutron sensitive material, ^10^B, is present in a thin layer on the inner wall of the proportional counter, making it sensitive to neutrons. The α-particle and the ^7^Li atom released in (2) are emitted from the boron layer in opposite directions. Due to this geometrical constrain only one of these species is, at best, emitted in the direction of the proportional gas, preventing the full collection of *Q* (Fig. 2).

While travelling inside the ^10^B layer, the α-particle and the ^7^Li lose a fraction of their initial energy, which is not collected in the proportional gas. As a consequence the output pulses of these detectors do not follow a Gaussian distribution as in ^3^He or BF_3_ proportional counters^5^,^9^. Instead, their pulse-height distributions are the sum of two flat distributions, each corresponding to one of the products released in (2). The result is a characteristic 2-step plateau distribution^8^,^9^,^10^,^11^ with maximal energy of 1.47 MeV and extending down to 0 (Fig. 2). This feature may represent a drawback for applications where a strong, low-energy gamma-ray background (from which the neutron must be discriminated) is present^6^. Another drawback of the neutron conversion in a ^10^B layer is that increasing the layer thickness only increases the detection efficiency up to a certain value: after some depth of interaction the α-particle and ^7^Li lose all their energy inside the boron layer, leaving the neutron un-accounted. In practice, the maximum detection efficiency achievable with a boron-lined proportional counter is roughly 12%^10^.

In this work we propose an alternative which combines the larger energy released in the ^10^B reaction with the full energy collection of ^3^He proportional counters. For that we have dispersed B_4_C nanoparticles in a proportional counter gas. The nanoparticles have dimensions smaller than the ranges of the α-particle and the ^7^Li in B_4_C, 3.6 and 1.8 μm, respectively^12^. In this situation an incoming neutron is converted inside a nanoparticle and both products of reaction (2) escape, depositing a large fraction of their energy in the proportional gas. Comparing to the case of a proportional counter filled with ^3^He and another gas, this solution corresponds to replacing the ^3^He atoms by B_4_C nanoparticles. The presence of the particles in the gas should not affect the properties of the proportional counter, either causing discharges or modifying its energy resolution. Under these conditions the detector response will present a full energy peak similar to the one of ^3^He proportional counters, but located at 2.3 MeV instead of 0.7 MeV.

## Results

**Figure 3:**
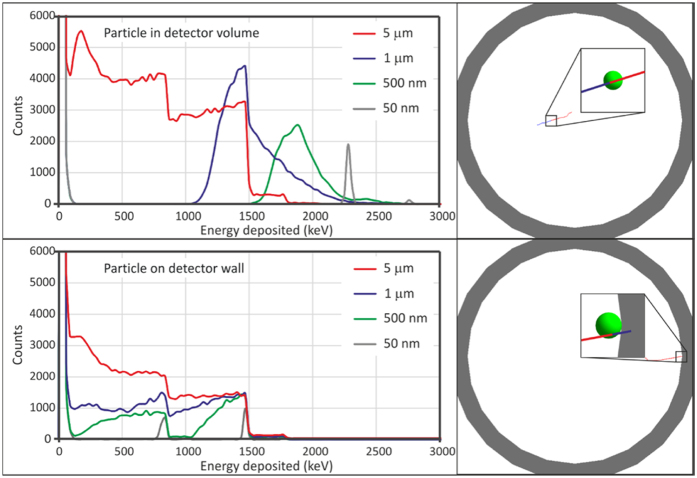
Results of the Monte Carlo simulation for a single B_4_C particle inside a proportional counter, for different particle radii. Top panel: left - B_4_C particle not in contact with the detector walls, energy deposited in the proportional gas by the ^7^Li and the α-particle; right - visualization of a neutron (not depicted) interacting in a 2-micron diameter B_4_C particle (green) with production of an α-particle (red) and ^7^Li (blue). Bottom panel: left - particle in contact with the walls of the detector, either the ^7^Li or the α-particle deposit energy in the proportional gas; right - visualization of a neutron (not depicted) interacting in a 2-micron diameter B_4_C particle (green) attached to the chamber walls (the ^7^Li is absorbed by the wall).

Prior to the experimental developments, we have performed a Monte Carlo simulation, comprising a single B_4_C particle inside a proportional counter filled with Ar-CH_4_ (90–10%). The simulation was implemented using GEANT4^13^ and 2 particle positions were studied: near the centre of the proportional counter (Fig. 3–Top panel) and in direct contact with the proportional counter walls (Fig. 3-Bottom panel). For each position, the particle radius was changed and the energy deposited in the proportional gas was recorded. The results show that, in the case of the particle not in contact with the detector walls (Fig. 3–Top panel) and for particle radii of 1 μm or lower, the response is indeed a single peak. For particles with a radius of 5 μm (Fig. 3–Top panel) or attached to the detector walls (Fig. 3-Bottom panel), the output is similar to the one of boron-lined proportional counters with maximal energy deposition of 1.47 MeV (for the 94% preferred branch in reaction 2). This is either due to self-absorption inside a larger particle (5 μm radius, Fig. 3–Top panel) or due to one of the secondary products being emitted onto the detector wall (particles attached to the walls, irrespective of their radius, Fig. 3-Bottom panel).

In order to evaluate the practicality of the proposed technique we have developed a test apparatus (methods section) composed of a proportional counter, filled with Ar-CH_4_ (90–10%), in which we have dispersed B_4_C nano-particles. During detector operation the gas circulates from the bottom to the top of the proportional counter, supporting the dispersion of the nanoparticles. A potential unwanted contribution to detector response could arise from nanoparticles attached to the walls of the proportional counter. In order to minimize such contribution we have equipped it with a set of electrodes (electric field gate), installed in the vicinity of its wall and surrounding the anode wire. This gate (methods section) allows establishing a reversed electric field in the region near the wall of the proportional chamber, preventing neutron interactions in nanoparticles attached to it from being detected.

**Figure 4:**
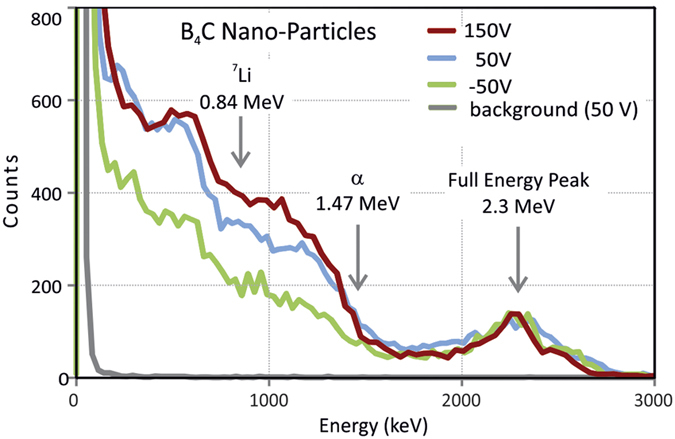
Pulse-height distributions of neutron induced pulses recorded with B_4_C nanoparticles, for different gate biasing voltages. Pulse-height distributions are normalized to the full energy peak. Red: V_GATE_ = 150 V. Blue: V_GATE_ = 50 V. Green: V_GATE_ = −50 V. Grey: V_GATE_ = 50 V, neutron beam off (background). Gate blocking efficiency for −50 V was 0.62. V_ANODE_ = 1850 V. Acquisition time = 400 sec. Neutron flux was 7 × 10^3^ n/sec.

Figure 4 presents the results of the irradiation of the prototype to a cold neutron beam line (Narzsiss Instrument, Paul Scherrer Institute, Switzerland), for several gate polarizations. Beside the 2-step plateau, limited by the ^7^Li and α-particle edges, the pulse-height distributions of Fig. 4 also present a single peak, with an energy corresponding to the sum of the α-particle and ^7^Li edges, representing full energy collection in the proportional gas.

## Discussion

For V_GATE_ = +150 V in Fig. 4, there is no blocking effect by the gate and the α-particle and ^7^Li edges are clearly identifiable in the pulse-height distributions. These features and the full-energy peak have been used for energy calibration of the pulse-height distributions of Fig. 4. As V_GATE_ is decreased to values below the ground potential, a reverse electric field is established in the region between the gate and the wall, which partially blocks interactions from material on the walls. This increases the relevance of the full-energy peak in the pulse-height distributions of Fig. 4. Nevertheless, for V_GATE_ = −50 V, a residual 2-step plateau is present in the pulse-height distributions. This may be due to attachment of nanoparticles to the electrodes of the gate, to residual interactions on the walls or to interactions near the gate.

The response of the detector to irradiation by the neutron beam indicates that some of the nanoparticles were dispersed in the proportional gas, resulting in full collection of the energy released in the neutron capture reaction. Such feature was, until now, only possible using either ^3^He or BF_3_ proportional counters. While the former has become increasingly unavailable, the latter is toxic and not suitable for applications outside a controlled environment. In addition, the energy emitted in the neutron reaction capture in ^10^B is higher (2.3 MeV) than that released with ^3^He (0.7 MeV), improving gamma ray discrimination.

A detection efficiency of 4% was achieved for V_GATE_ = −50 V. This figure is related with the total volume of the aerosol, the nanoparticles concentration and their composition. All these parameters can, in principle, be improved. In particular, the use of ^10^B enriched nanoparticles will increase the detection efficiency in the same proportion as the enrichment ratio. The electric field gate was successful in improving the pulse-height distributions; however, a technical challenge remains in decreasing the attachment of particles to the chamber wall and further increasing the full-energy peak relevance. Fluids other than gases, such as room temperature^14^ or cryogenic liquids^15^,^16^, are also candidates to disperse the nanoparticles.

This novel technique uses a non-toxic and abundant material and transforms a regular proportional counter gas into a neutron sensitive aerosol. The type of response recorded is an improvement over state-of-the-art-alternatives^6^,^7^,^8^,^10^,^11^ with events in which the total energy released in the neutron capture reaction is collected. The detection of neutrons with nanoparticles immersed in a gas constitutes a breakthrough in the field of radiation detection and has the potential to become an alternative for neutron detection.

## Methods

**Figure 5:**
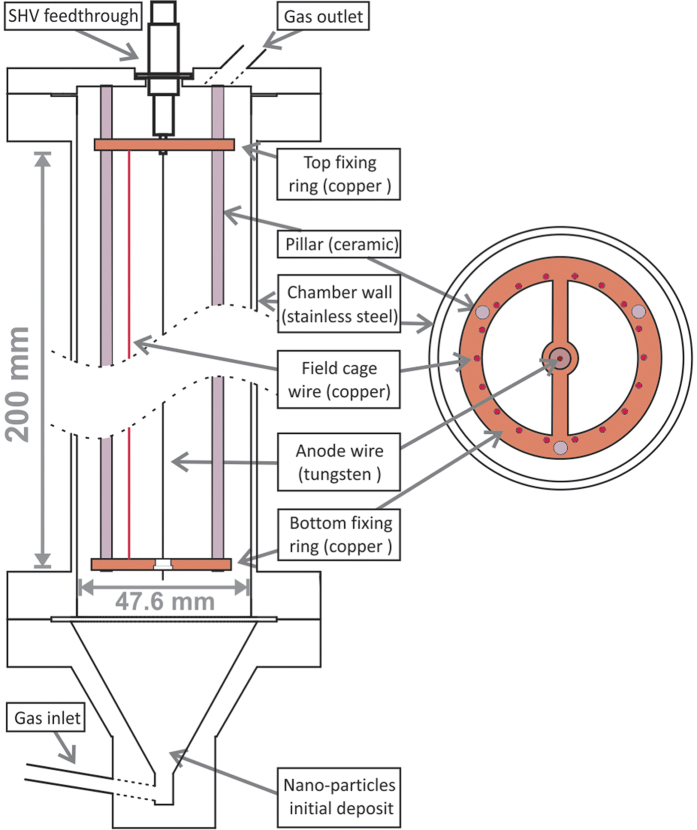
Left: Schematics of the proportional counter used in this work. Three ceramic pillars, glued to the top flange of the detector, serve as support for the top and bottom fixing rings. The anode wire was soldered to the SHV connector on the top flange, stretched and fixed to the centre of the bottom ring. Only one of the 18 field cage wires is depicted. Right: cross section view. The positions of the 18 field cage wires are depicted, as well as the bottom fixing ring, where the anode wire is glued to.

The main component of the experimental setup is a custom-made gaseous proportional counter (Fig. 5). It is composed of a stainless steel cylinder with an inner diameter of 47.6 mm and a 50 μm diameter tungsten wire stretched along its axis. The tungsten wire (anode) is electrically insulated from the outer cylinder (cathode), which is connected to ground potential. One end of the anode wire is soldered to an SHV feedthrough while the other end is glued to a ceramic insulator on the bottom fixing ring. The bottom and top fixing rings are supported by 3 ceramic pillars, glued to the top flange. When the anode is biased with a positive voltage (1850 V were used throughout the measurements presented in this work), a radial electric field is established inside the chamber. This electric field is responsible for collecting the primary electrons converted by the ionizing radiation in the proportional gas and for drifting them towards the anode. Once the primary electrons reach a small region in the vicinity of the anode, where the electric field is higher than the ionization threshold of the gas, they undergo a proportional multiplication process by impact ionization with the gas molecules. Multiplication factors above 10^3^ are commonly achieved in proportional counters^17^. In our apparatus the resulting electron charge was collected at the anode with a charge-sensitive pre-amplifier (Canberra 2006) which fed a linear amplifier (Ortec 454). A multichannel analyser digitized the output pulses from the amplifier.

During detector operation a mixture of Ar-CH_4_ (90–10%) was continuously flowing throughout the main chamber at a rate of 4 l/h, supplied from an adjacent pressurized canister. Gas admission into the chamber was achieved through a ¼ inch entrance located at the bottom of the chamber (gas inlet in Fig. 5). The consumed gas leaved the chamber through a ¼ inch outlet, located at the top of the chamber. This outlet was equipped with a 2-μm filter (Swagelok NI-4-VCR-2-GR-2M) and was insulated from the outer atmosphere by a gas bubbler filled with vacuum pump oil.

The stainless steel chamber was placed vertically and the particles to be dispersed were loaded into the bottom flange, immediately after the gas inlet. Prior to detector operation, the proportional gas mixture was first allowed to flow for one hour at a rate of 0.8 l/h, to ensure that the atmospheric air was removed from the inside of the stainless steel cylinder. Afterwards, with the purpose of facilitating particle dispersion, the flow was increased for a short period to values above 8 l/h. Data taking was done at a constant flow of 4 l/h.

The apparatus was installed in the Swiss Spallation Neutron Source (SINQ) at the Narziss beam line, which provides a continuous beam of cold neutrons (5 Å). The neutron beam, with vertical and horizontal dimensions of 50 mm and 10 mm, respectively, irradiated the apparatus in the centre of the stainless steel cylinder, in a direction perpendicular to its axis, at equal distances from the top and bottom flanges. With such beam collimator settings the beamline intensity was of 7 × 10^3^ neutrons per second. A beam blocker in the neutron beam line allowed turning on and off the neutron beam.

Uncoated B_4_C nanoparticles (PlasmaChem GmbH, B_4_C Nanopowder) were used for the measurements presented in this paper. A sample was dispersed in ethanol and its particle size distribution measured by laser diffraction (Beckman Coulter LS 13 320). A mean value of 1.056 μm with d_10_ = 0.553 μm, d_50_ = 1.029 μm and d_90_ = 1.602 μm was measured. During data taking the detector was stable, without the occurrence of any electrical discharge or large gain fluctuations.

Eighteen copper wires (0.8 mm in diameter) were installed inside the stainless steel chamber, at a distance of 10 mm from the walls and in a direction parallel to the anode wire (Fig. 5, right). The polarization of this set of electrodes (electrostatic gate) with a negative potential established an electric field between the gate and the wall of the stainless steel chamber. Such electric field configuration should prevent the neutron conversions taking place in the material attached to (or near) the wall to be detected. The range in Ar-CH_4_ (90–10%) of the α-particle emitted in reaction (2) was calculated to be 8.7 mm^18^. Taking this information into account, the set of electrodes was placed at a distance of 10 mm from the walls. The gate blocking efficiency to neutron conversions taking place next to the walls of our chamber was determined in a former experimental measurement. For that, a thin slab of boron silicone was attached to the inner walls of the chamber. The detector was exposed to the Narziss beam line and the pulse-height distributions were recorded for several values of V_GATE_. For V_GATE_ = −50V the blocking efficiency (relative to V_GATE_ = 150 V) was measured, being 62%.

The Monte Carlo simulation (Fig. 4) was implemented with the GEANT4 software package^13^, version 9.5. With an extensive set of physics models and cross section database, this tool allows handling the interactions of particles with matter across a very wide energy range being used to accurately simulate the passage of particles through matter. For the simulation a standard physics list (EmStandard PhysicsList) was used and the main physical components of the experimental apparatus was reproduced, simulating its response to thermal neutrons, for different positions and diameters of a single B_4_C particle. A total of 10^7^ thermal neutrons were generated for each simulation, emitted in the direction of the particle, and uniformly distributed over its cross section. For each event, the simulation records the energy deposited in the proportional counter gas.

## Additional Information

**How to cite this article**: Amaro, F. D. *et al*. Novel concept for neutron detection: proportional counter filled with ^10^B nanoparticle aerosol. *Sci. Rep.* **7**, 41699; doi: 10.1038/srep41699 (2017).

**Publisher's note:** Springer Nature remains neutral with regard to jurisdictional claims in published maps and institutional affiliations.

## Figures and Tables

**Figure 1:**
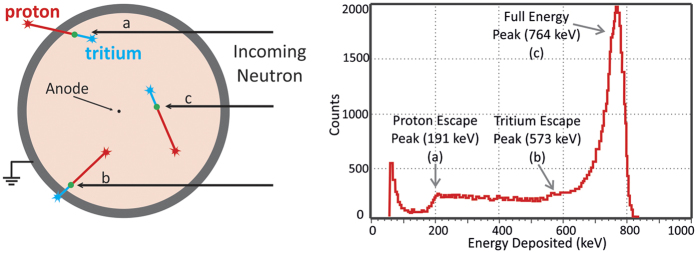
Left: Neutron interaction in ^3^He proportional counters: an incoming neutron interacts with a ^3^He atom, producing a proton (573 keV) and a tritium (191 keV). In large detectors most of the interactions are of type c) and result in full energy (764 keV) collection. For interactions near the walls there is a probability that one of the products is emitted onto the wall of the detector and its energy is not collected, resulting in the low energy continuum with 2 discontinuities at 191 and 573 keV. Right: pulse-heigh distribution recorded with a ^3^He proportional counter, adapted from^5^.

**Figure 2:**
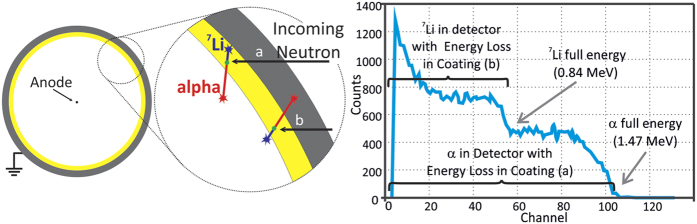
Left: Neutron interaction in boron-10 lined proportional counter. Possible outcomes of a neutron interaction with a boron-10 atom of the layer: (**a**) α-particle is emitted in the direction of the proportional gas, with energy loss in the boron-10 layer; (**b**) ^7^Li is emitted in the proportional gas, with energy loss in the boron-10 layer. Right: Pulse-height distribution recorded with a boron-10 linned proportional counter, adapted from^10^. Interactions of type (**a**) produce events with energies up to the α-particle threshold. Super-imposed on these are the type (**b**) events, with maximum energy corresponding to the ^7^Li energy.
