# Corrigendum: Novel concept for neutron detection: proportional counter filled with ^10^B nanoparticle aerosol

**DOI:** 10.1038/srep46942

**Published:** 2018-02-12

**Authors:** F. D. Amaro, C. M. B. Monteiro, J. M. F. dos Santos, A. Antognini

Scientific Reports 7: Article number: 4169910.1038/srep41699; published online: 02 09 2017; updated: 02 12 2018

The Acknowledgements section in this Article is incomplete.

“F.D.A. and C.M.B.M. acknowledge the support of FCT, under contracts SFRH/BPD/74775/2010 and SFRH/BPD/76842/2011, respectively. We acknowledge R. Ferreira (UCQfarma), J. Catita and J. Carvalheira (Paralab), for their assistance with the particle size distribution measurement. The measurements were performed at the Swiss Spallation Neutron Source SINQ at the Paul Scherrer Institute. We acknowledge C. Schanzer of the SwissNeutronics AG and M. Horisberger for access to the neutron beam.”

should read:

“F.D.A. and C.M.B.M. acknowledge the support of FCT, under contracts SFRH/BPD/74775/2010 and SFRH/BPD/76842/2011, respectively. We acknowledge R. Ferreira (UCQfarma), J. Catita and J. Carvalheira (Paralab), for their assistance with the particle size distribution measurement. The measurements were performed at the Swiss Spallation Neutron Source SINQ at the Paul Scherrer Institute. We acknowledge C. Schanzer of the SwissNeutronics AG and M. Horisberger for access to the neutron beam. This work is financed by national funds thru FCT – Fundação para a Ciência e a Tecnologia, I.P., in the framework of project UID/FIS/04559/2013.”

